# Does additional support provided through e-mail or SMS in a Web-based Social Marketing program improve children’s food consumption? A Randomized Controlled Trial

**DOI:** 10.1186/s12937-018-0334-1

**Published:** 2018-02-16

**Authors:** Natalie Rangelov, Sara Della Bella, Pedro Marques-Vidal, L. Suzanne Suggs

**Affiliations:** 1 BeCHANGE Research Group, Institute of Public Communication, Università della Svizzera italiana, Via G. Buffi 4, 6900 Lugano, Switzerland; 2 Department of Medicine, Internal Medicine, Lausanne University Hospital (CHUV), Rue du Bugnon 46, 1011 Lausanne, Switzerland; 3 Institute of Global Health Innovation, Faculty of Medicine, Imperial College London, London, UK

**Keywords:** Nutrition, Children, RCT, Intervention, Web, E-mail, SMS, E-health, Eating behavior, Social marketing

## Abstract

**Background:**

The FAN Social Marketing program was developed to improve dietary and physical activity habits of families with children in Ticino, Switzerland. The aim of this study was to examine if the effects of the program on children’s food intake differed by intervention group.

**Methods:**

Effects of the FAN program were tested through a Randomized Controlled Trial. The program lasted 8 weeks, during which participants received tailored communication about nutrition and physical activity. Families were randomly allocated to one of three groups, where the parent received the intervention by the Web (G1), Web + e-mail (G2) or Web + SMS (G3). Children in all groups received tailored print letters by post. Children’s food consumption was assessed at baseline and immediate post intervention using a 7-day food diary. Generalized linear mixed models with child as a random effect and with time, treatment group, and the time by treatment interaction as fixed effects were used to test the impact of the intervention.

**Results:**

Analyses were conducted with a sample of 608 children. After participating in FAN the marginal means of daily consumption of fruit changed from 0.95 to 1.12 in G1, from 0.82 to 0.94 in G2, and from 0.93 to 1.18 in G3. The margins of the daily consumption of sweets decreased in each group (1.67 to 1.56 in G1, 1.71 to 1.49 in G2, and 1.72 to 1.62 in G3). The change in vegetable consumption observed from pre to post intervention in G3 (from 1.13 to 1.21) was significantly different from that observed in G1 (from 1.21 to 1.17).

**Conclusions:**

A well-designed Web-based Social Marketing intervention complemented with print letters can help improve children’s consumption of water, fruit, soft drinks, and sweets. The use of SMS to support greater behavior change, in addition to Web-based communication, resulted only in a small significant positive change for vegetables, while the use of e-mail in addition to Web did not result in any significant difference.

**Trial registration:**

The trial was retrospectively registered in the ISRCTN registry (ID ISRCTN48730279).

**Electronic supplementary material:**

The online version of this article (10.1186/s12937-018-0334-1) contains supplementary material, which is available to authorized users.

## Background

There is low adherence to nutritional guidelines in both adults and children in Switzerland. Among the adult population, only 19% of men and women consume the recommended five portions of fruit and vegetables each day [1], and the average meat consumption (780 g per week per person) far exceeds the recommended amount of 240 g per week [2, 3]. Swiss children also do not adhere to the recommended guidelines with about 55% of girls and 40% of boys eating fruits and vegetables daily [4]. A study conducted in Canton Ticino, one of the 26 States in Switzerland, showed that less than 50% of the children were adherent to the national dietary guidelines [5]. Looking at fruit consumption, only 10.4% of children adhered to the recommendations. No child consumed the recommended amount of vegetables, and only 9.5% adhered to the guidelines for soft drinks, sweets and salty snacks (the others over-consumed those foods). Children in Ticino are also over-consumers of meat (72.7%) [5]. Further, Swiss data also show high rates of overweight and obesity: more than 40% of adults and roughly 20% of children are overweight or obese in Switzerland [6–9]. At the time of the study, compared to the rest of Switzerland, Canton Ticino presented the highest rates for overweight and obesity for adults (39.9%) and for children (23%) [10–13]. As dietary habits acquired during childhood persist into adult life and are leading factors for many health issues [14, 15], it is important to promote a healthy diet to children.

Health programs designed to influence children’s diet are quite heterogeneous in that they have been conducted in different settings (schools, homes, communities), used different study designs (cohort studies, Randomized Controlled Trials), were informed by different theories (e.g., Theory of Planned Behavior, Social Cognitive Theory) and followed different approaches (for instance Social Marketing or health promotion). Interventions aimed at changing children’s food consumption have often involved parents, who, as role models and providers of food, exert a powerful influence on children’s food consumption [16–18].

Social Marketing is a framework that integrates Marketing principles with other approaches to promote healthy behaviors, with the final aim of benefiting society. Social Marketing focuses on behavior and integrates best practice, theory, research, and a deep population analysis to develop effective behavior change interventions [19]. Social Marketing is the approach that is recommended by the World Health Organization to promote healthy nutrition and other lifestyle behaviors related to risks for non–communicable diseases [20, 21]. Reviews of Social Marketing studies suggests that Social Marketing has been effectively used to change health-related behaviors [22–24] and in promoting a healthier diet [24, 25]. The review conducted by Carins & Rundle-Thiele of Social Marketing studies for healthy eating showed that the majority of examined studies achieved positive behavior change. Further, the review showed that several healthy eating behaviors were improved, including fruit, vegetable, fat, and water intake [25].

Information and Communication Technology based programs for nutrition and healthy weight promotion can have positive effects in prompting and supporting behavior change [26–29]. In particular, there is evidence that Web-based interventions are effective in changing behavior [30–33]. Further, Short Messaging Service (SMS) and e-mails have been used as reminders and cues to action to improve engagement with interventions and to reinforce behavior change [26, 29]. Results from a systematic review by Hutchesson and colleagues (2015) showed that obesity prevention e-interventions targeting different behaviors (i.e. nutrition, physical activity, weight maintenance) are primarily delivered through Websites, but that e-mail, SMS and other phone applications are increasingly being tested in isolation or in conjunction [26]. Another systematic review showed that using additional communication beyond a Web-only intervention increased the effectiveness of Web-based interventions, with SMS having a greater impact than e-mails [29].

Still, it is not clear to what extent SMS or e-mails directed to parents can improve children’s eating behavior above and beyond a Web-based intervention. The aim of this study was thus to examine the effect of a Social Marketing healthy nutrition program on children’s food intake. We aimed to assess if additional support parents received through e-mail or SMS resulted into additional behavior change of their child over that of the Web-only group. The primary outcome was change in children’s food consumption from pre- to post- intervention, according to intervention group.

The following hypotheses were tested:

1. Healthy food consumption would increase in all groups
2. Unhealthy food consumption would decrease in all groups
3. The e-mail group would show greater improvement than the Web-only group
4. The SMS group would show greater improvement than the Web-only group

## Methods

The Web-based Social Marketing program called FAN “Famiglia, Attività fisica, Nutrizione” was designed to promote a healthy food consumption and regular physical activity among families living in Ticino, Switzerland [34, 35]. To develop FAN, the Social Marketing benchmarks (citizen orientation; behavior; theory; insight; exchange value; competition; segmentation; and methods mix) [36] were considered and followed [34, 35]. The methods mix includes the marketing mix: product, place, price, promotion, policy and partnership. All six were included in the development of FAN. Formative research was conducted with the target population (both children and their parents) to get to know and better understand their needs, but also their wants, regarding the content and the strategies for an intervention promoting healthy food consumption and physical activity. In particular, results of the focus groups and interviews conducted with the parents showed that while they used all three technologies daily (Web, e-mail, SMS), they were more keen to receive a Web-based intervention, compared to an e-mail or SMS intervention. Being very busy, they welcomed this approach, that would allow them to take the intervention anytime and anywhere, at their convenience. They also approved of e-mail and SMS, but only in case we used them with parsimony. Hence, we developed a Web-based intervention, using SMS and e-mail as reminders. Further details about the development of the study can be found in Rangelov and Suggs (2015) [34].

To be eligible to participate, families had to a) live in Ticino; b) be able to complete surveys in Italian; c) have Internet access, an e-mail address, and a mobile phone; and d) have a child attending primary school, or first two grades of secondary school. The program was offered free of charge. Study procedures were reviewed by the Canton Ticino Ethics Committee and deemed exempt in accordance with Swiss law. In accordance with the recommendations of the Helsinki Declaration, both children and parents provided informed consent and voluntarily provided their data.

FAN was funded by the Ticino Department of Health and Social Affairs and Health Promotion Switzerland (see Additional file 1 CONSORT Checklist) and all eligible families willing to participate were allowed to enroll. Parents were invited by the FAN team through a brochure and information letter distributed to children in all but four elementary and all middle schools of Canton Ticino between June 15th and September 15th 2010. Enrollment required two steps. First, families registered through the FAN Website (edizione1.fanticino.ch), providing their consent, contact information, their gender, the number of children, and their children’s gender and grade at school. The baseline (BL) survey was sent to all those registered (see further information below) and had to be completed in the week of 13th–19th September. Parents that completed the BL were randomly assigned to one of three groups using Excel random draw command; Web-only (group G1), Web + e-mail (group G2) or Web + SMS (group G3).

The intervention lasted 8 weeks, during which children and their parents received tailored information regarding nutrition and physical activity behaviors. Content was tailored based on gender of the parent, number and gender of the children, and based on the behavior that was perceived as being the most difficult to perform (physical activity or nutrition). All the delivered content was based on pre-existing material used in the Canton, and repackaged for the various communication channels used in this study. Every week, parents received new content on the password-protected Website and children received a personalized and tailored letter by post.

The Website was updated every Tuesday morning with a new theme related to nutrition. For example, the first week, titled “You are off to a good start and that is half the battle!” provided information about the recommendations for a healthy diet, while in the fifth week, theme called “Lunch and snacks with imagination”, suggestions on how to cope with the lack of time to cook at lunch time and ideas for quick and healthy lunches and snacks were presented. Beyond providing information about the importance of healthy nutrition, the Website provided practical advice including recipes and tips on how to eat better, how to introduce healthier food to the family, and how to deal with concerns. Content was shown in form of short text, pictures, and videos. A forum was also available to families where they could discuss things together and with a dietician.

The e-mails and SMS were used as weekly reminders to prompt parents in G2 and G3 to visit the Website. In addition to providing a link to the Website, the e-mail provided a short summary of the weekly theme, in form of a short text, a main image on top of the text, and links to different pages on the Website at the bottom. For example, the text of the e-mail in the fifth week was: “Dear Ms. Rossi, Welcome back to FAN! This week we would like to show you some alternatives for a healthy lunch. […] Has it ever happened that you ate a sandwich or a hamburger, because it was easier, because you did not have time or because you did not find another healthier alternative? […] We suggest you a healthy recipe that is easy to prepare. The ingredients are: salad, tuna fish, olive oil, and… find the rest on the Website [link]! […]”.

The SMS also included a link to the Website, along with a message aimed to stimulate motivation or provide support (e.g. “You do not have much time to cook over lunch time, but you’d like that your children eat healthy? Visit the FAN Website!”). These were sent every Tuesday morning, after the Website was updated. Further details about the communication and behavior change strategies can be found in Rangelov and Suggs (2015) [34].

After excluding children that were not eligible, and those that did not receive the allocated program, 735 children were included in the study. Since for technical problems some families did not receive the allocated intervention, these families were reallocated to the correct group. For instance, 14 families allocated to G1 also received an e-mail: these families were reallocated to G2. Thirty-one families allocated in G2 did not receive the e-mail for technical issues; hence they were reallocated to G1. Finally, three families in G3 did not receive any SMS (issues with their mobile phone) and were reallocated to G1, and 18 families received the e-mail and the SMS, hence they were excluded from analyses.

**Fig. 1:**
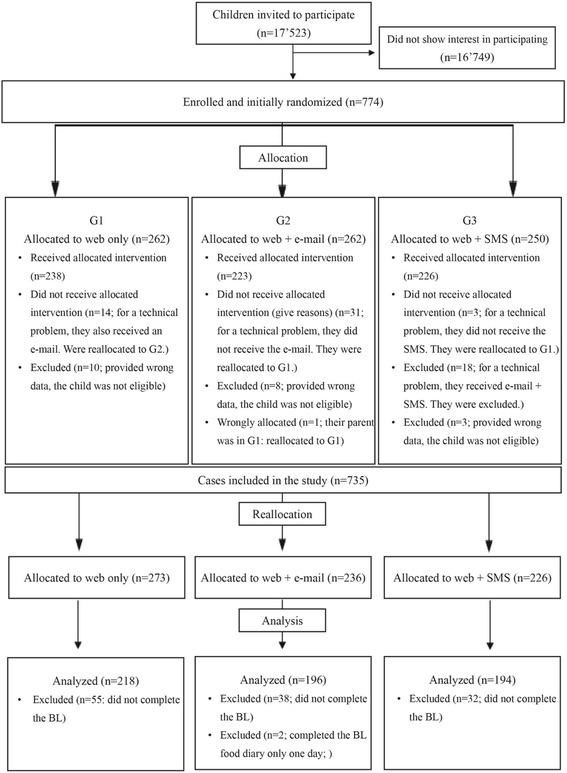
CONSORT Flow Diagram

No parent or child withdrew from the program. Of those, 125 children were excluded from analyses as they did not complete the BL, and two were excluded as they completed the BL food diary only 1 day. The final sample included 452 parents and 608 children, divided as follows: G1) Web-only (*n* = 163 parents; *n* = 218 children), G2) Web + e-mail (*n* = 144 parents, *n* = 196 children), and G3) Web + SMS (*n* = 145 parents; *n* = 194 children). There were 308 parents participating with one child, 133 parents with two children, 10 parents with three children and one parent with four. All children of the same parent were placed in the same group as their parent (for further details, see Fig. 1 CONSORT Flow Diagram). Since Web-based programs have shown to be effective in other studies and the program was available to all eligible families as part of the funding agreement, the Web-only group (G1) served as the control group, and the e-mail (G2) and SMS (G3) groups were expected to produce additional benefits.

Gender, age, height and weight of the children were collected at baseline (BL) through a print survey completed by parents. Height and weight were used to calculate body mass index (BMI). Age and gender-specific BMI cutoffs from the U.S. Centers for Disease Control and Prevention, validated for Swiss children, were applied [37]. Food intake data were collected from children at both BL and at follow up (FUP - November 29th – December 5th). For each day of the week, children reported what they ate using a 7-day food diary [38]. The 7-day food diary was tested in another study by the same authors to compare agreement between children and their parents, showing that children are reliable food reporters [38]. Based on the Swiss Society for Nutrition (SSN) [39], 12 food categories were coded: water; fruit (fresh, dried or baked; 100% fruit juice); vegetables (fresh and cooked, also vegetable soup; 100% vegetable juice); starchy foods; meat; fish; eggs; dairy products (i.e. milk, cheese, yoghurt); fats (i.e. oil, butter, olives, nuts); fat meat and fat fish (i.e. salami, breaded fried meat or fish); sweets (i.e. cookies, jam, cakes, chocolate, ice-cream); and soft drinks (i.e. fizzy soft drinks, sweetened ice-tea, sweetened still juices, syrups). Frequency of consumption of each food was recorded, and the mean of frequency of consumption was used as mean of daily intake [38]. Portion sizes were not recorded as children of this age range have been shown to be unreliable in accurately quantifying their food intake [40–42]. All data were entered in a database and double-checked to limit data entry errors. The database was stored on the University server, accessible only to the research team.

Bivariate comparisons were performed using one-way ANOVA for continuous variables and χ^2^-test for categorical variables. K-Wallis test was used to compare the median frequency of consumption of selected food categories at baseline across groups. Intention to treat analyses were performed. The effect on food consumption for each intervention group was assessed using mixed models with pre- and post- intervention data. The models included a main effect (time) that measures the effect of the intervention on the control group (i.e. the Web-only group) and an interaction term (time*group) which indicates whether the effect of the intervention varied in the interventions groups (SMS and e-mail groups) compared to the control one. Daily frequency of consumption was analyzed using linear mixed models with child as a random effect and with time, treatment group, and the time by treatment interaction as fixed effects. Adequacy of the models was assessed by creating QQ-plots for the residuals of first and second level and that for all items (see Additional file 2: Figures S1-S4). Except for fish, eggs and sugar drinks no clear violation of the normality assumption was found. Analyses were performed with cases that completed the food diary for at least 4 days out of seven at BL. A sensitivity analysis was performed including only participants who completed the food dairy for at least 4 days at both BL and FUP.

At the time of the study, there were no data available regarding food consumption among children in Ticino, nor information about possible effects of a Social Marketing intervention similar to ours on food consumption in children. Further, as this was an intervention at the cantonal level and in real-life setting, we could not limit our sample, nor we could make the intervention mandatory to participants. Hence, we could not conduct a power analysis prior to the study, and we had to rely on the available sample size to conduct the analyses. The sample size was assessed post-hoc based on the results of the study and an alpha value of 5% and a power of 80% were used.

All analyses were conducted using Stata version 14.1 (Stata Corp, College Station, TX, USA). All tests were two-sided and considered significant at the *p* < .05 level.

## Results

The mean age of children was 8.5 (SD = 1.9) and 49.3% were boys. The baseline characteristics and food consumption of the children are presented in Tables 1 and 2, respectively. The groups did not differ at BL (see Table 2).

**Table 1:** Children’s characteristics at baseline (full sample and by intervention group)

Characteristics at baseline	Total (*N* = 608)	Group 1 Web-only (*n* = 218)	Group 2 Web + e-mail (*n* = 196)	Group 3 Web + SMS (*n* = 194)	*p*-value
Boys (%)	49.3	51.4	44.9	51.5	0.319
Age (years)	8.5 (1.9)	8.4 (1.9)	8.7 (1.9)	8.4 (1.9)	0.170
	(*N* = 588)	(*n* = 215)	(*n* = 184)	(*n* = 189)	
BMI (%)					0.060
Underweight	10.4	14.8	8.1	7.4	
Healthy-weight	72.1	67.9	76.6	72.5	
Overweight or obese	17.5	17.2	15.2	20.1	

*SD* Standard Deviation. *BMI* Body Mass Index. Results are expressed as column percentage for categorical variables or as mean (standard deviation) for continuous variables. Between-group comparisons performed using χ2-tests for categorical variables and ANOVA for continuous variables

**Table 2:** Daily frequency of consumption at baseline by intervention group

	Full sample (*N* = 608)	Group 1 Web-only (*n* = 218)	Group 2 Web + e-mail (*n* = 196)	Group 3 Web + SMS (*n* = 194)	*p*-value
FOC [median (first-third quartile)]					
Water	1,57 [0,43–2,14]	1,57 [0,43–2,14]	1,71 [0,71–2,14]	1,57 [0,43–2,14]	0.63
Fruit	0,86 [0,43–1,29]	0,86 [0,43–1,43]	0,71 [0,43–1,14]	0,86 [0,43–1,43]	0.08
Vegetables	1,14 [0,86–1,43]	1,29 [1–1,57]	1,14 [0,86–1,43]	1,14 [0,86–1,43]	0.15
Starches	2,71 [2,29–3,14]	2,71 [2,29–3]	2,71 [2,29–3,14]	2,86 [2,43–3,14]	0.06
Meat	0,71 [0,57–1]	0,71 [0,57–0,86]	0,71 [0,57–1]	0,71 [0,57–0,86]	0.75
Fish	0,14 [0–0,29]	0,14 [0–0,29]	0,14 [0–0,29]	0,14 [0–0,29]	0.07
Eggs	0,14 [0–0,29]	0,14 [0–0,29]	0,14 [0–0,29]	0,14 [0–0,29]	0.99
Dairy products	1,71 [1,29–2,14]	1,71 [1,43–2]	1,71 [1,29–2,14]	1,79 [1,29–2,14]	0.43
Fat	0,86 [0,57–1,14]	0,71 [0,57–1]	0,86 [0,43–1]	0,86 [0,57–1,14]	0.55
Fat meat/fat fish	0,29 [0,14–0,43]	0,14 [0,14–0,43]	0,29 [0,14–0,43]	0,29 [0,14–0,43]	0.55
Sweets	1,71 [1,29–2,14]	1,71 [1,29–2]	1,71 [1,29–2,14]	1,71 [1,29–2,14]	0.88
Soft drinks	0,43 [0,14–0,86]	0,43 [0,14–0,86]	0,43 [0,14–0,86]	0,29 [0,14–0,86]	0.60

*FOC* frequency of consumption. Results presented as median (first-third quartile). Between-group comparisons performed using K-Wallis test was used to compare groups

The Website registered a total of 22′559 visits over the 8 weeks of the intervention [43]. It was visited by a total of 195 parents with 261 children (G1) 72 parents with 95 children; G2) 56 parents with 78 children; and G3) 67 parents with 88 children) (see Table 3). When children were asked whether they themselves visited the Website, 39% in G1, 30% in G2 and 39% in G3 answered that they did.

**Table 3:** Frequency and percentage of parents who visited the FAN Website and of children whose parents visited the Website

	Group 1 Freq. (%)		Group 2 Freq. (%)		Group 3 Freq. (%)	
	Parents	Children	Parents	Children	Parents	Children
Visited the Website (*n* = 195 parents)	72 (44.17%)	95 (43.58%)	56 (38.89%)	78 (39.80%)	67 (46.21%)	88 (45.36%)
All (*N* = 452 parents)	163 (100%)	218 (100%)	144 (100%)	196 (100%)	145 (100%)	194 (100%)

*P* Parents, *C* Children

The marginal means of daily consumption of food and change between BL and FUP for the full sample and by group are presented in Table 4. The results of the generalized linear models estimating the effect of the intervention are presented in Table **5**. The frequency of consumption of fruit significantly increased (+ 0.17) in G1 from BL to FUP. G2 and G3 did not significantly differ from G1 in terms of change in fruit consumption. The daily frequency of consumption of sweets decreased significantly by 0.11 in G1 (Table 4). G2 and G3 did not significantly differ from G1 in terms of change in sweets consumption between BL and FUP. The frequency of vegetable consumption did not change significantly from BL to FUP for any group. However, the change from BL to FUP in G3 significantly differed (+ 0.08) compared to the change in G1 (− 0.04). The intervention did not have other significant effects.

**Table 4:** Margins/marginal means (Std. Err.) of daily consumption of food of children

	Full sample (*N* = 608)			Group 1 Web-only (n = 218)			Group 2 Web + e-mail (n = 196)			Group 3 Web + SMS (n = 194)		
	BL	FUP	change	BL	FUP	change	BL	FUP	change	BL	FUP	change
Water	1.49 (0.05)	1.50 (0.06)	+ 0.01	1.42 (0.08)	1.45 (0.10)	+ 0.03	1.54 (0.08)	1.50 (0.11)	+ 0.04	1.51 (0.09)	1.57 (0.11)	+ 0.06
Fruit	0.90 (0.03)	1.08 (0.04)	+ 0.18	0.95 (0.05)	1.12 (0.08)	+ 0.17	0.82 (0.05)	0.94 (0.06)	+ 0.12	0.93 (0.06)	1.18 (0.07)	+ 0.25
Vegetables	1.17 (0.02)	1.16 (0.03)	− 0.01	1.21 (0.03)	1.17 (0.04)	−0.04	1.16 (0.04)	1.09 (0.06)	−0.07	1.13 (0.04)	1.21 (0.04)	+ 0.08
Starchy foods	2.72 (0.03)	2.63 (0.03)	−0.09	2.68 (0.05)	2.62 (0.05)	−0.06	2.68 (0.04)	2.63 (0.06)	−0.05	2.79 (0.05)	2.66 (0.05)	−0.13
Meat	0.74 (0.01)	0.74 (0.02)	0.00	0.73 (0.02)	0.70 (0.03)	−0.03	0.75 (0.03)	0.77 (0.03)	+ 0.02	0.76 (0.02)	0.74 (0.03)	−0.02
Fish	0.17 (0.01)	0.16 (0.01)	−0.01	0.19 (0.01)	0.17 (0.01)	−0.02	0.18 (0.01)	0.16 (0.02)	−0.02	0.15 (0.01)	0.16 (0.02)	+ 0.01
Eggs	0.15 (0.01)	0.14 (0.01)	−0.01	0.15 (0.01)	0.13 (0.01)	−0.03	0.15 (0.01)	0.16 (0.02)	+ 0.01	0.15 (0.01)	0.13 (0.01)	−0.02
Dairy products	1.71 (0.03)	1.73 (0.03)	+ 0.02	1.67 (0.04)	1.71 (0.06)	+ 0.04	1.75 (0.06)	1.77 (0.06)	+ 0.02	1.71 (0.05)	1.72 (0.05)	+ 0.01
Fat	0.84 (0.02)	0.92 (0.03)	+ 0.08	0.82 (0.03)	0.90 (0.05)	+ 0.07	0.82 (0.04)	0.91 (0.04)	+ 0.09	0.88 (0.04)	0.97 (0.05)	+ 0.09
Fat meat/fat fish	0.27 (0.01)	0.27 (0.01)	0.00	0.25 (0.02)	0.28 (0.02)	+ 0.03	0.30 (0.02)	0.28 (0.04)	−0.02	0.26 (0.02)	0.26 (0.02)	0.00
Sweets	1.70 (0.03)	1.55 (0.03)	−0.15	1.67 (0.04)	1.56 (0.05)	−0.11	1.71 (0.05)	1.49 (0.07)	−0.22	1.72 (0.05)	1.62 (0.06)	−0.10
Soft drinks	0.57 (0.03)	0.44 (0.03)	−0.13	0.56 (0.04)	0.45 (0.07)	−0.11	0.59 (0.06)	0.46 (0.05)	−0.13	0.55 (0.05)	0.41 (0.05)	−0.14

**Table 5:** Effect of the intervention on daily consumption of food by group of intervention

Food categories	Group 1 Web-only (*n* = 218)	Group 2 Web + e-mail (vs control) (*n* = 196)	Group 3 Web + SMS (vs control) (*n* = 194)
Water	0.03 (0.09)	−0.07 (0.12)	0.02 (0.12)
Fruit	0.17* (0.07)	−0.06 (0.09)	0.08 (0.09)
Vegetables	−0.04 (0.04)	−0.03 (0.06)	0.12* (0.06)
Starchy foods	−0.06 (0.05)	−0.01 (0.07)	− 0.07 (0.07)
Meat	−0.03 (0.03)	0.05 (0.05)	0.01 (0.05)
Fish	−0.02 (0.02)	0.00 (0.02)	0.03 (0.03)
Eggs	−0.03 (0.02)	0.03 (0.02)	0.01 (0.02)
Dairy products	0.04 (0.05)	−0.02 (0.07)	−0.03 (0.07)
Fat	0.07 (0.06)	−0.01 (0.07)	0.01 (0.07)
Fat meat/fat fish	0.03 (0.03)	−0.05 (0.05)	−0.03 (0.03)
Sweets	−0.11* (0.05)	−0.11 (0.07)	0.00 (0.08)
Soft drinks	−0.11 (0.06)	−0.02 (0.08)	− 0.04 (0.08)

Results are presented as regression coefficients (Std. Errors)

*Significant at the *p* < .05 level

### Sensitivity analysis

Roughly 47% of children in G1, 43% in G2, and 41% in G3 did not complete the FUP or completed it for less than 4 days. Results of the sensitivity analysis show that children who did not complete the FUP, or completed it for less than 4 days, and children who completed the food dairy for at least 4 days out of seven at both BL and FUP were comparable in terms of BL characteristics (see Table 6), and food consumption. Children with complete data only differ in terms of a higher intake of fruit and dairy at BL. The effects of the intervention were also similar across the two samples.

**Table 6:** Baseline characteristics of children with complete data (at least 4/7 days at both BL and FUP) and children with incomplete data (less than 4/7 days at FUP)

	Children with complete data (*n* = 341)	Children with incomplete data (*n* = 267)	*p*-values
Gender (%)			
Boys	51.3	46.8	0.270
Mean Age (SD)	8.5 (1.9)	8.5 (1.9)	0.793
	Children with complete data (*n* = 333)	Children with incomplete data (*n* = 255)	
BMI (%)			0.337
Underweight	9.3	11.8	
Healthy-weight	74.5	69.0	
Overweight or obese	16.2	19.2	
	Children with complete data (*n* = 341)	Children with incomplete data (*n* = 267)	
FOC [median (first-third quartile)]			
Water	1,71 [0,57–2,14]	1,57 [0,43–2,14]	0.150
Fruit	0,86 [0,43–1,43]	0,71 [0,29–1,29]	0.049
Vegetables	1,14 [0,86–1,57]	1,14 [0,86–1,43]	0.342
Starchy foods	2,71 [2,43–3,14]	2,57 [2,29–3,14]	0.012
Meat	0,71 [0,57–0,86]	0,71 [0,57–1]	0.525
Fish	0,14 [0–0,29]	0,14 [0–0,29]	0.177
Eggs	0,14 [0–0,29]	0,14 [0–0,29]	0.952
Dairy products	1,86 [1,43–2,14]	1,71 [1,29–2]	< 0.001
Fat	0,86 [0,57–1,14]	0,71 [0,57–1]	0.135
Fat meat/fat fish	0,29 [0,14–0,43]	0,14 [0,14–0,43]	0.131
Sweets	1,71 [1,29–2,14]	1,57 [1,29–2]	0.025
Soft drinks	0,43 [0,14–1]	0,29 [0,14–0,71]	0.007*

*FOC* frequency of consumption. Results presented as means plus standard deviation (SD). T-test for continuous variables. *BMI* Body Mass Index. *BL* Baseline assessment. *FUP* Follow-up assessment

## Discussion

The aim of this study was to test whether FAN had a positive effect on children’s food consumption, and whether the effect of the FAN program differed by treatment group. The hypotheses were that children would increase their consumption of healthy food (H1), they would decrease their consumption of unhealthy food (H2), and that children whose parents received weekly e-mails (G2) or SMS (G3) prompts, in addition to the Web intervention, would show more positive outcomes than G1 (H3 and H4). This study is unique in that the Web-intervention was for the parent and the behavior measured was that of the child.

Overall, the intervention effects were not different across groups. Children increased their daily consumption of fruit, and decreased that of sweets regardless of the group they were assigned. For example, if we consider fruit consumption, we can see that fruit consumption of children in G1 increased by 0.17 times/day at FUP, which means, at least one more fruit consumption per day. Children in G2 and G3 also increased their fruit consumption, even if this increase was not statistically significant (0.12 in G2 and by 0.25 for G3, summing the effect of the Web and that of e-mail or SMS respectively). This means, for instance, that if at BL children ate fruit only at one occasion per day (FOC = 1), after the intervention they ate it 0.17 times per day (G1), 1.12 times (G2) and 1.25 times a day (G3), meaning at least an additional occasion of consumption per day, which is meaningful from a public health perspective. The effects of vegetable consumption were positive for those children whose parents received SMS prompts and were negative for G1 and G2, suggesting that to improve vegetable consumption, SMS prompts help. While not significant, the results obtained for G2 show that the e-mail had a positive effect on fat meat and fat fish, which consumption decreased, compared to G1 where it increased and to G3 where it did not change. Compared to G3, the effects in G2 for fruit, water, and soft drinks were slightly smaller, while they were larger for sweets.

A systematic review by Webb and colleagues (2010) including studies on several health topics (physical activity and nutrition among others), shows evidence to support the use of e-mail and SMS in addition to Web content [29]. They found that using those additional modes of delivery increased the effects on behavior change, and that SMS use had larger effects, compared to e-mail use [29]. Our results suggest that adding SMS reminders to parental communication only resulted in a small positive effect on the consumption of vegetables. Since only 39% of the parents in G2 and 46% in G3 visited the Website, it is possible that without the reminders the rates would have been even lower. As this is the first study, to our knowledge, that includes a tailored Web-based component for parents and a tailored print letter sent directly to children, further research is warranted.

### Strengths and limitations

This is the first study that was conducted with children and their parents in Ticino, Switzerland, and that assessed children’s food consumption using a RCT design based on the communication delivered to parents. This fills the gap regarding the lack of available data about food consumption among children in Ticino, and it provides insight regarding to what extent SMS or e-mails directed to parents can improve children’s eating behavior above and beyond a Web-based intervention.

Further, the aim of this study was to examine the effect of a Social Marketing healthy nutrition program on children’s food intake. The results show that children increased their daily consumption of fruit, and decreased that of sweets, which is an important finding for public health. Indeed, food consumption habits are among the leading factors for many health issues, and increasing fruit and decreasing sweets consumption are among the recommendations for a healthy diet. Since the Social Marketing framework was used to develop the FAN intervention, and since positive results were found, FAN could be easily adapted to other Cantons in Switzerland, or other countries, with the aim of improving children’s nutrition.

The sample was homogeneous in terms of baseline characteristics and analyses showed similar results when comparing children with complete data to children with incomplete data. However, a possible limitation is the sample size. Indeed, the power calculations suggested that a much bigger sample size was needed to detect meaningful differences in food consumption change between three groups (e.g. for fruit, N of 2544 per intervention group would be needed; for soft drinks *N* = 3244 per group). This would require sampling approximately one fourth of the entire child population aged 6–12 of the canton, which would require considerable financial and logistic resources. Also, the sample might not be fully representative of the Ticino child population: we compared the gender and age distribution of our sample to the corresponding distribution in the canton of Ticino, obtained from the canton’s statistical office [44]. No differences were found regarding age; conversely, the study sample had a higher prevalence of 6-year olds and a lower prevalence of children aged 11 and older (Additional file 3: Table S1).

Model adequation could not be fully ascertained for fish, eggs and sugar drinks, as normality of the first or second level residuals could not be fully assessed. Still, GLMM are sufficiently robust to handle small deviations from normality and homoscedasticity.

The effects found might be due to the combination of the communication to the parent and the communication to the child, or solely due to the communication sent to the child. Future research should examine if interventions effects differ when the communication is sent to the child only, compared to parent only, and to both parent and child.

Moreover, it may be that the effects were partly due to other causes and not to the intervention per se. For example, children may have become more aware of their food choices and changed their behavior simply because they were asked to indicate what they ate. However, this is common in these types of studies, as noted by Macdiarmid and Blundell (1998) [45], and using a 7-day food diary allowed the accurate collection of data, while minimizing observation effects [38].

## Conclusions

A Social Marketing tailored program for parents delivered through the Web and complemented with tailored letters directed to children might be enough to improve children’s consumption of water, fruit, soft drinks and sweets. The beneficial use of e-mail and SMS to support greater behavior change beyond Web-based communication is mixed. In the case of vegetable consumption, sending additional support through SMS to parents may be worth the investment.

## Additional files

Additional file 1: CONSORT 2010 checklist. (DOC 217 kb)

Additional file 2: QQ-plots for the Residuals of First and Second Level for all Food Items. (ZIP 416 kb)

Additional file 3: Comparison between the FAN and Ticino Samples. (DOCX 12 kb)

## Abbreviations

BL: Baseline
FAN: Famiglia, Attività fisica, Nutrizione (Family, physical Activity, Nutrition)
FUP: Follow up
G1: Group 1: Web-only
G2: Group 2: Web + e-mail
G3: Group 3: Web + SMS
RCT: Randomized Controlled Trial
SMS: Short Messaging Service
