# Supplementary material for: Does additional support provided through e-mail or SMS in a Web-based Social Marketing program improve children’s food consumption? A Randomized Controlled Trial
Source: Nutr J. 2018 Feb 16;17:24. doi: 10.1186/s12937-018-0334-1 (PMC5815187; doi:10.1186/s12937-018-0334-1)

**Supplementary Figure 3**

QQ-plots for the residuals of first and second level for eggs, dairies and fat.

## Eggs

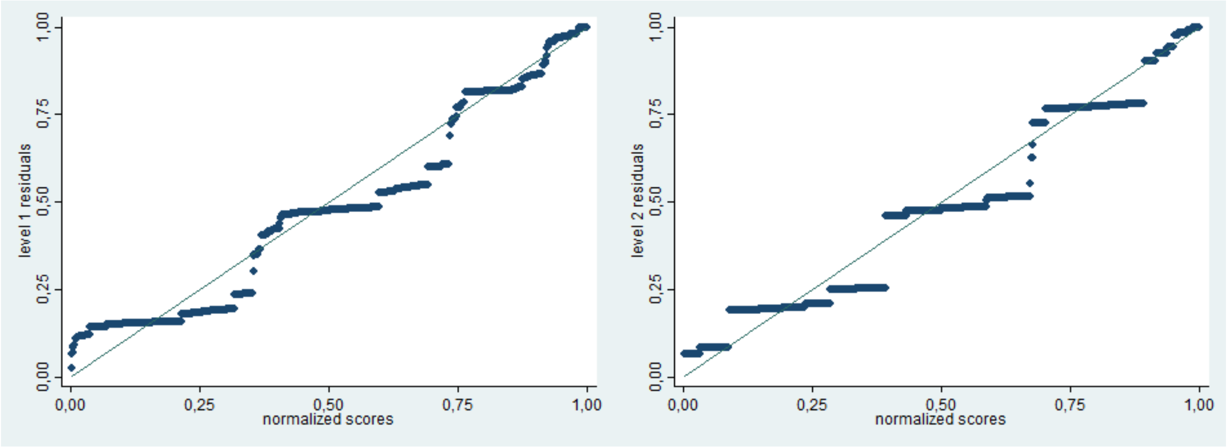


## Dairies

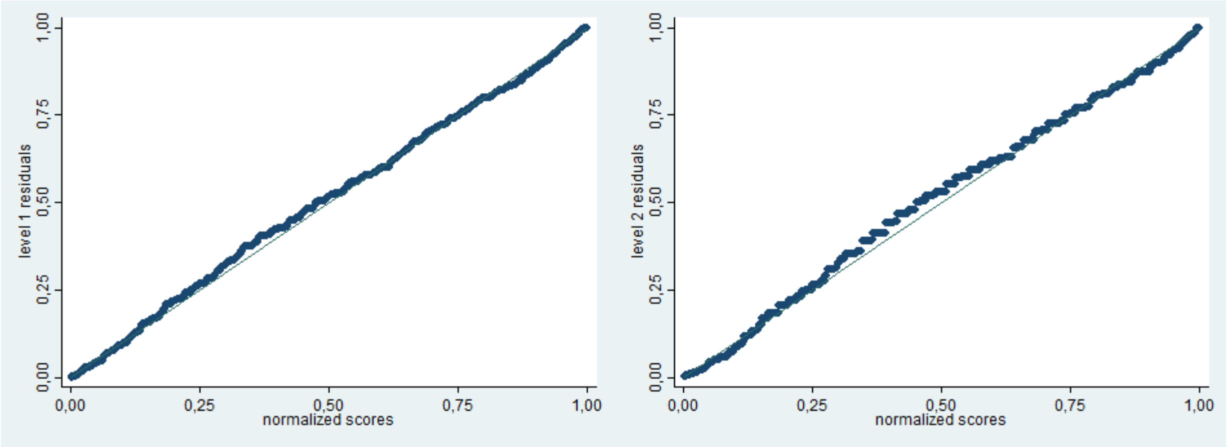


## Fat

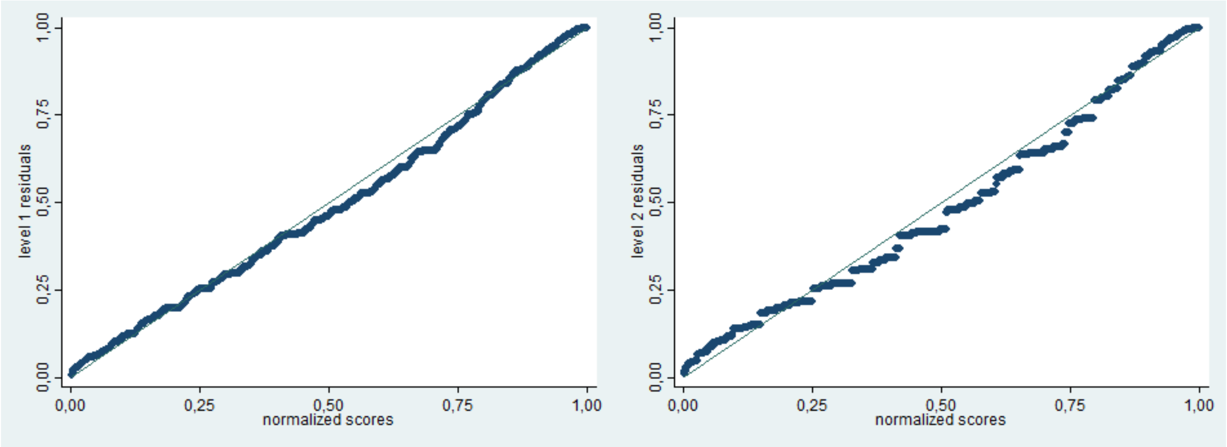

Supplement: Supplementary file 2 — QQ-plots for the Residuals of First and Second Level for all Food Items. (ZIP 416 kb) [file 12937_2018_334_MOESM2_ESM.zip › Supplementary Figure 3_eggs, dairies, fatR4.pdf]
