# Supplementary material for: Does additional support provided through e-mail or SMS in a Web-based Social Marketing program improve children’s food consumption? A Randomized Controlled Trial
Source: Nutr J. 2018 Feb 16;17:24. doi: 10.1186/s12937-018-0334-1 (PMC5815187; doi:10.1186/s12937-018-0334-1)
Supplement: Supplementary file 3 — Comparison between the FAN and Ticino Samples. (DOCX 12 kb) [file 12937_2018_334_MOESM3_ESM.docx]

**Supplementary Table 1.**

Comparison between the FAN and Ticino Samples

|  | **FAN** | | **Ticino** | |
| --- | --- | --- | --- | --- |
| **Age (years)** | Freq. | % | Freq. | % |
| 6 | 122 | 20.17 | 3006 | 13.69 |
| 7 | 94 | 15.54 | 2978 | 13.56 |
| 8 | 106 | 17.52 | 3147 | 14.33 |
| 9 | 91 | 15.04 | 3002 | 13.67 |
| 10 | 93 | 15.37 | 3306 | 15.06 |
| 11 | 57 | 9.42 | 3218 | 14.66 |
| 12 | 42 | 6.94 | 3299 | 15.03 |
